# Percutaneous renal sympathetic denervation combined with empirical Purkinje de-networking for electrical storm in congenital long QT syndrome after cardiac sympathetic denervation: a case report with two consecutive patients

**DOI:** 10.1007/s10840-026-02324-8

**Published:** 2026-04-23

**Authors:** Tolga Aksu, Emrah Ermiş, Ahmad Huraibat, Mehdi Onac, Mohamed Niang, Mehmet Akif Vatankulu, Hakan Ucar, Henry D. Huang

**Affiliations:** 1 Department of Cardiology, MLPCARE, Medical Park Florya Hospital, İstanbul Aydin University, Istanbul, 34742 Turkey; 2 Division of Cardiology, Chicago Universityi , Chicago, IL 60612 USA

**Keywords:** Long QT syndrome, Electrical storm, Ventricular fibrillation, Purkinje de-networking, Renal sympathetic denervation, Cardiac sympathetic denervation

## Abstract

Electrical storm in congenital long QT syndrome (LQTS) may persist despite maximal β‑blockade, implantable cardioverter-defibrillator (ICD) therapy, and surgical cardiac sympathetic denervation (SCSD). When no reproducible premature ventricular contractions are documented, catheter ablation targets and procedural endpoints are uncertain. We report two consecutive young adults (LQTS1 and LQTS2) with recurrent ventricular fibrillation and appropriate ICD shocks despite prior left SCSD. In both, sinus-rhythm left ventricular substrate mapping showed no scar or abnormal electrograms. A mechanistic strategy was therefore selected: empirical mapping and elimination of Purkinje potentials along the distal left anterior and posterior fascicles (“Purkinje de-networking”), followed by percutaneous renal sympathetic denervation in the same session. Programmed stimulation with and without isoproterenol was non-inducible after ablation, and both patients remained free of ventricular arrhythmia recurrence and ICD therapies during follow-up (16 and 8 months) on β‑blocker therapy. This combined, percutaneous bailout approach may be considered in highly selected LQTS patients with electrical storm refractory to standard therapies.

## Introduction

Congenital long QT syndrome (LQTS) is an inherited cardiac channelopathy associated with polymorphic ventricular tachycardia, ventricular fibrillation (VF), and sudden cardiac death in structurally normal hearts [1]. β‑blockers are first-line therapy, yet a subset of symptomatic patients experience recurrent arrhythmic events and appropriate intracardiac defibrillator (ICD) therapies [2]. Left surgical cardiac sympathetic denervation (SCSD) can reduce arrhythmic burden, but electrical storm may persist in rare, highly refractory cases [3, 4]. Renal sympathetic denervation (RDN) has been used as an adjunct neuromodulatory strategy in malignant ventricular arrhythmias, and Purkinje fibers are recognized triggers/maintainers of VF in diverse clinical contexts [5–8]. However, an established catheter ablation strategy for LQTS-related VF is lacking, particularly when no consistent triggering premature ventricular contractions (PVCs) are captured. We describe two consecutive LQTS patients with electrical storm despite β‑blockade, ICD protection, and prior left SCSD who were treated with a single-session percutaneous dual approach: empirical Purkinje de-networking plus RDN.

## Case presentation

A 24-year-old man with genetically confirmed LQTS type 1 (KCNQ1) had a VVI ICD (Medtronic, Minneapolis, MN, USA) implanted 7 years earlier for recurrent syncope despite propranolol (4 mg/kg/day). He underwent left SCSD 3 years before presentation because of recurrent appropriate ICD shocks. Transthoracic echocardiography demonstrated structurally normal hearts with preserved left ventricular systolic function and no significant valvular disease.

**Fig. 1:**
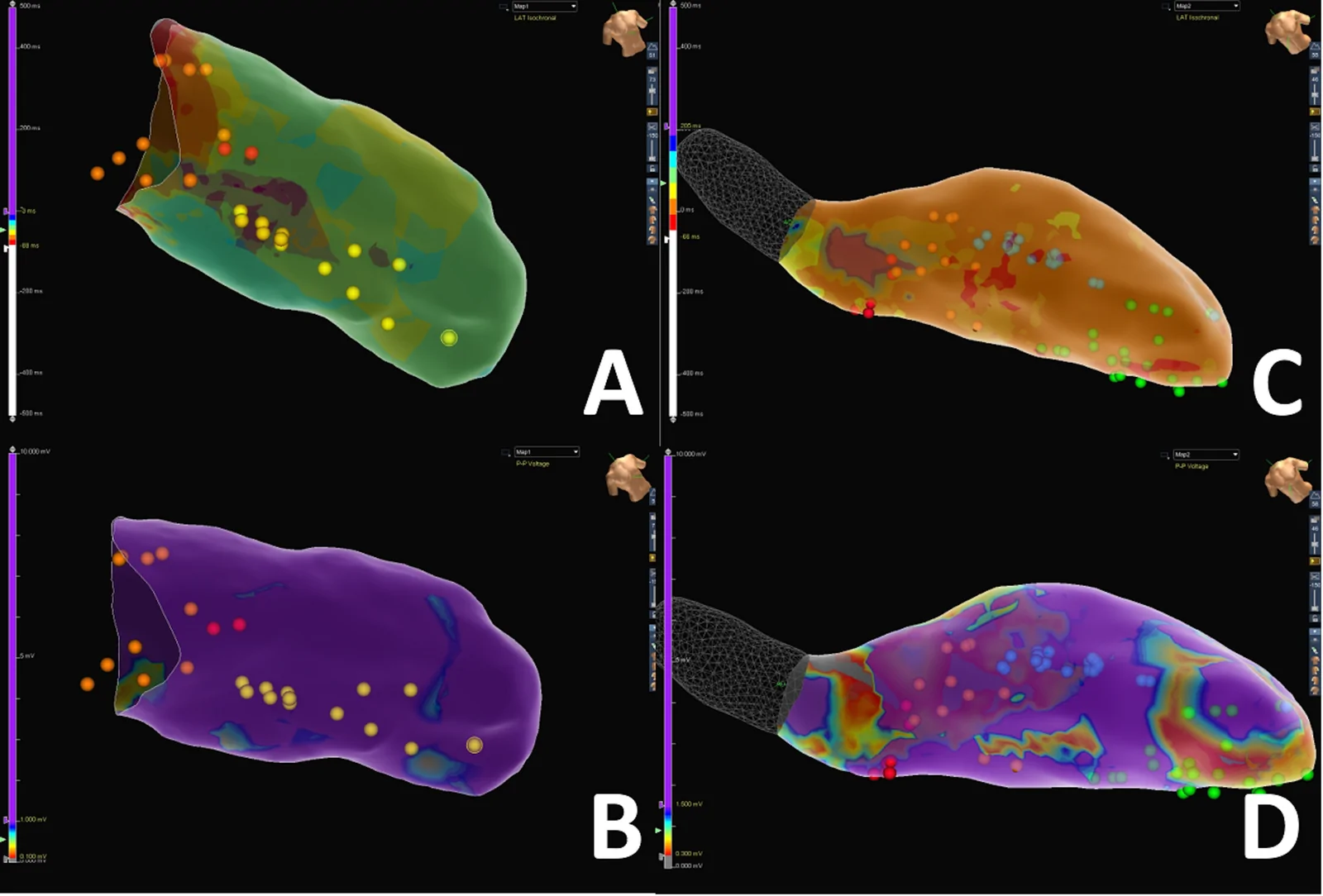
Three-dimensional isochronal late activation (**A**,** C**) and voltage (**B**, **D**) electroanatomic maps of patient 1 and 2, respectively. The left bundle branch potentials were marked by orange tag points. His potentials were tagged by red tag points. Purkinje potentials were marked by yellow, blue, and green tag points. Ablation was targeted to the Purkinje arborization along the distal left anterior and posterior fascicles (approximately LV segments 3–5), aiming to eliminate all visualized Purkinje potentials

Over the month preceding admission, VF episodes were triggered by progressively less exertion, culminating in three appropriate shocks within 24 h. Intravenous β‑blocker and magnesium were administered, and the patient stabilized. No consistent PVCs were identified on telemetry or during electrophysiological study. Programmed ventricular stimulation from standard sites failed to induce sustained ventricular arrhythmia. Endocardial left ventricular voltage and electrogram mapping (HD Grid catheter with EnSite™ system) revealed no low-voltage regions, late potentials, or abnormal electrograms. Given the absence of a focal trigger and a negative substrate map, the left-sided conduction system was systematically mapped and Purkinje potentials were tagged along the distal left anterior and posterior fascicles (including the dead-end tract and lateral papillary muscle region) (Fig. 1A, B). Radiofrequency energy was delivered to eliminate identifiable Purkinje potentials (“de-networking”).

**Fig. 2:**
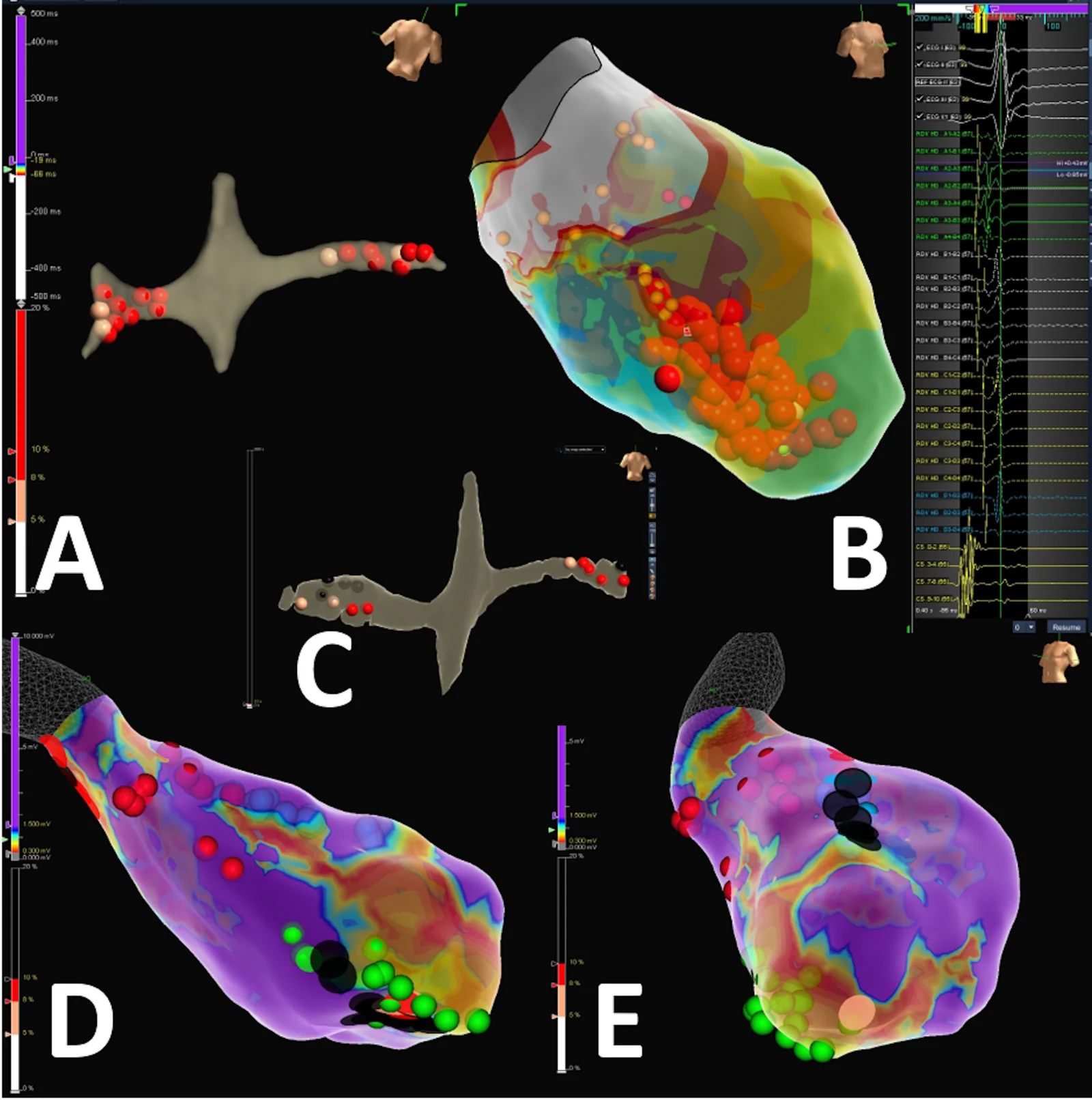
Electroanatomical mapping and ablation lesion distribution in two patients. Panels **A** and **B** illustrate findings from the first patient. Panel **A** shows the electroanatomical map of the renal artery, highlighting the locations of ablation lesions applied along the arterial wall. Panel **B** demonstrates the left ventricular electroanatomical map, with clustered ablation lesions targeting the arrhythmogenic region within the left ventricle. Panels **C**, **D**, and **E** correspond to the second patient. Panel **C** depicts the renal artery ablation lesions, illustrating the distribution and extent of energy delivery along the renal artery. Panels **D** and **E** show different views of the left ventricular electroanatomical maps, demonstrating the spatial distribution of ablation lesions within the left ventricle. Color-coded voltage maps and lesion tags are used to represent myocardial substrate characteristics and ablation sites in both patients. Right panel shows Purkinje potentials by a multipolar diagnostic catheter (HD Grid, Abbott) in above

Immediately thereafter, percutaneous RDN was performed using a standard trans-femoral approach. After selective renal angiography, radiofrequency applications were delivered circumferentially in both renal arteries (15 W, 43 °C, 20 s per application; total 17 applications) (Fig. 2A, B). Post-procedure programmed stimulation with and without isoproterenol was non-inducible. Renal function remained unchanged and office blood pressure decreased by approximately 10–15 mmHg systolic without symptomatic hypotension, measured during routine clinical follow-up. At 16 months, ICD interrogation showed no recurrent VF and no ICD therapies while the patient continued propranolol.

**Fig. 3:**
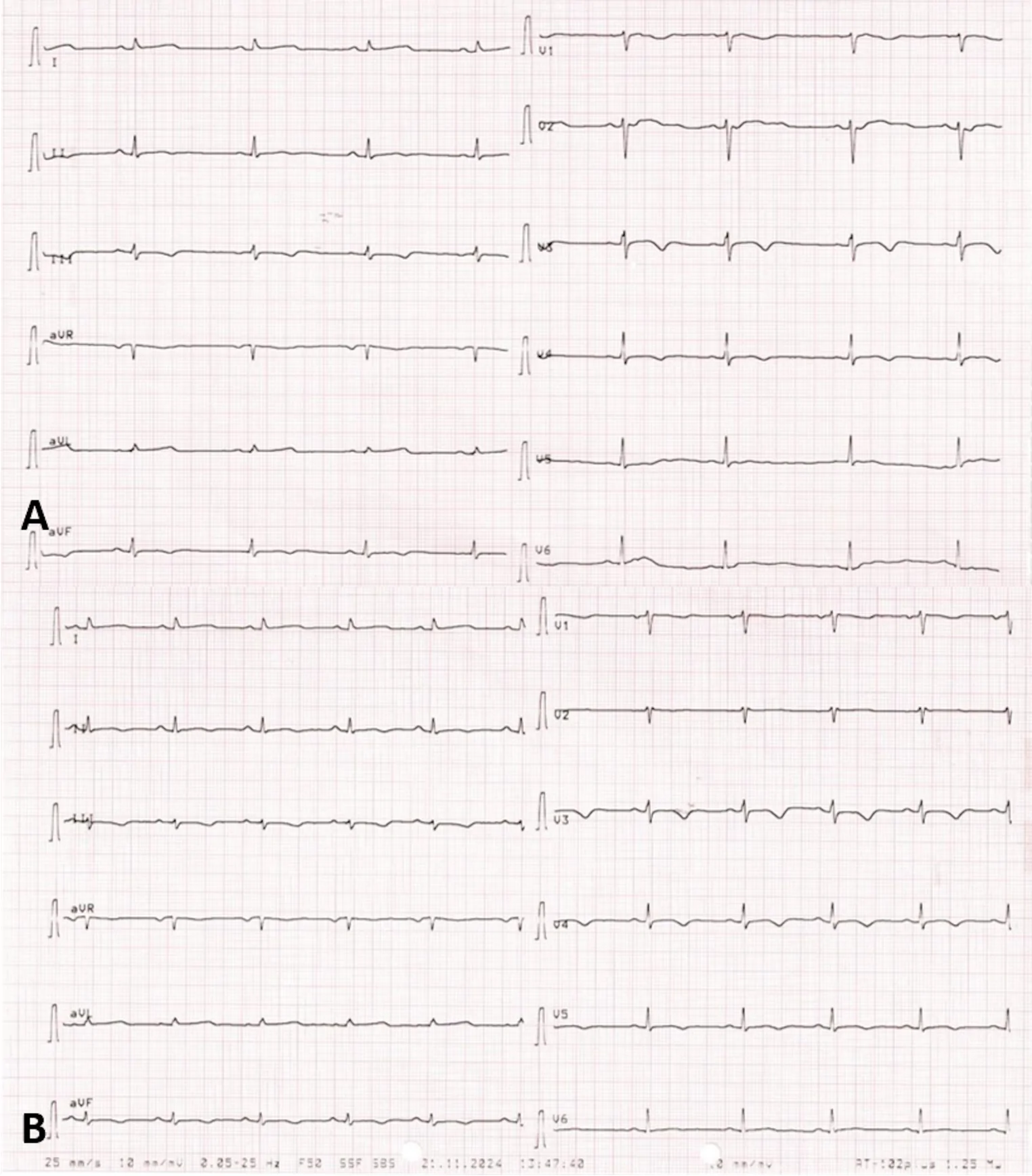
Pre- and post-procedural 12-lead electrocardiograms. Representative 12-lead electrocardiograms obtained before (**A**) and after (**B**) the combined procedure (empirical Purkinje de-networking and renal sympathetic denervation). The baseline ECG demonstrates congenital long QT syndrome–related repolarization abnormalities prior to intervention. The post-procedural ECG illustrates rhythm characteristics and repolarization pattern following ablation, without evidence of acute conduction disturbances. ECG recordings were acquired during sinus rhythm under stable clinical conditions

Patient 2: A 22-year-old woman with LQTS type 2 (KCNH2) had a VVI ICD (Medtronic, Minneapolis, MN, USA) implanted 5 years earlier for recurrent syncope despite propranolol (2 mg/kg/day) and underwent left SCSD 1 year earlier due to recurrent appropriate ICD shocks. She presented after two exercise-associated ICD shocks over 2 weeks. Echocardiography revealed normal cardiac structure and preserved LVEF. No PVCs were documented on Holter monitoring. Programmed stimulation was non-inducible, and endocardial left ventricular mapping with HD Grid catheter (EnSite™ system) again demonstrated no scar or abnormal electrograms. Purkinje potentials along the distal left anterior and posterior fascicles were mapped and eliminated with radiofrequency ablation, followed by bilateral RDN in the same session (15 applications, 15 W, 43 °C, 20 s) (Figs. 1C and D and 2C-E). Ventricular arrhythmias were non-inducible post-ablation. Pre- and post-procedure ECGs are seen in Fig. 3. At 8 months, ICD interrogation showed no recurrent VF or ICD therapies on continued propranolol.

## Discussion

These two consecutive cases illustrate a practical, mechanistic bailout strategy for malignant ventricular arrhythmias in congenital LQTS when conventional therapies fail. Both patients had recurrent VF/electrical storm despite β‑blockers, ICD protection, and prior SCSD, and neither demonstrated a reproducible triggering PVC nor an abnormal ventricular substrate on endocardial mapping. In this setting, focal trigger ablation is not feasible, and the operator is left with limited percutaneous options beyond additional neuromodulation or empirical substrate modification.

RDN may reduce systemic sympathetic outflow by interrupting afferent renal signaling and has been used as adjunct therapy in refractory ventricular arrhythmias, including a reported congenital LQTS case [5]. In parallel, the Purkinje network can serve as a final common pathway for VF initiation and perpetuation through focal activity or micro-reentry, even when a consistent trigger is not captured [6]. Empirical Purkinje de-networking therefore aims to reduce VF susceptibility by eliminating identifiable Purkinje potentials and disrupting potential propagation paths in otherwise normal myocardium [6–8].

Our procedural endpoints were (i) elimination of recordable Purkinje potentials in the targeted regions and (ii) non-inducibility with a reproducible programmed stimulation protocol performed before and after intervention, with and without isoproterenol. Although non-inducibility is an imperfect surrogate, it provides an immediate before/after assessment of arrhythmia vulnerability when spontaneous triggers are absent. Both patients remained free of recurrent VF and ICD therapies during follow-up while continuing β‑blockers.

This approach should be interpreted cautiously. First, the number of patients is small and the respective contributions of Purkinje de-networking and RDN cannot be separated. Second, epicardial mapping was not performed; therefore, an unrecognized epicardial substrate cannot be excluded [9]. Third, in this specific context, non-inducibility should be interpreted cautiously and was considered a procedural observation rather than a definitive endpoint; long-term arrhythmia freedom and absence of ICD therapies remain the clinically meaningful outcomes. Last, Haissaguerre et al. [10] has previously documented the importance of PVC coupling intervals, and VF cycle lengths as potentially helpful in determination of idiopathic VF without structural abnormality. Detailed PVC coupling interval or VF cycle length analysis was limited due to absence of consistent triggering ectopy in our cases. Contemporary EHRA/HRS expert consensus documents emphasize individualized multimodality approaches in refractory electrical storm, particularly when conventional therapies fail [11].

In highly selected LQTS patients with recurrent VF/electrical storm despite SCSD and optimal medical therapy, a percutaneous dual strategy that combines empirical Purkinje de-networking with RDN may represent a feasible option in experienced centers, ideally within a research framework and with careful multidisciplinary discussion.

## Data Availability

No datasets were generated or analysed during the current study.
